# A case report of prostate cancer with leptomeningeal metastasis and bone marrow involvement

**DOI:** 10.1002/cnr2.2041

**Published:** 2024-04-05

**Authors:** Maryam Garousi, Nima Mousavi Darzikolaee, Ali Faridfar, Seyed Mohammadreza Javadi, Esmaeil Samizadeh, Masoumeh Sajadi Rad, Reyhaneh Bayani

**Affiliations:** ^1^ Department of Radiation Oncology School of Medicine, Iran University of Medical Sciences Tehran Iran; ^2^ Radiation Oncology Research Center Cancer Institute, Tehran University of Medical Sciences Tehran Iran; ^3^ Department of Radiation Oncology Imam Khomeini Hospital Complex, School of medicine, Tehran University of Medical Sciences Tehran Iran; ^4^ Research Center for Cancer Screening and Epidemiology AJA University of Medical Sciences Tehran Iran; ^5^ General Surgery Department Hamadan University of Medical Sciences Hamadan Iran; ^6^ Department of pathology School of medicine AND Imam Reza Hospital, AJA University of Medical Sciences Tehran Iran

**Keywords:** leptomeningeal involvement, prostate cancer

## Abstract

**Background:**

Prostate cancer is the second most common cancer in men. Central nervous system (CNS) involvement in prostate cancer which manifests as cerebral, leptomeningeal, or dural involvement is uncommon and occurs late in the course of disease.

**Case:**

A 60‐year‐old patient with castration resistant prostate cancer (CRPC) presented with headache and fatigue. Evaluation revealed bone marrow and leptomeningeal involvement. The patient treated by whole brain radiotherapy, leuprolide, weekly docetaxel and daily 1000 mg abiraterone. Complete blood count (CBC) and CNS symptoms improved and the patient is alive after 11 months with excellent performance status.

**Conclusion:**

Leptomeningeal involvement in prostate cancer is rare and is associated with a poor prognosis but the possibility of such event should be considered in patients with new onset progressive CNS symptoms. New treatment strategies such as combination of docetaxel and abiraterone added to androgen deprivation therapy (triplet therapy) might improve outcome in these patients.

## 1 INTRODUCTION

Prostate cancer is the second most common cancer in men.^1^ Although prostate cancer mortality is mostly caused by extensive bone metastases but in few cases, it might be due to brain, liver or lung involvement. Central nervous system (CNS) involvement in prostate cancer, which manifests as cerebral, leptomeningeal, or dural involvement is uncommon and occurs late in the course of disease.^2^ New treatment strategies have increased survival in prostate cancer patients and prevalence of leptomeningeal involvement has been increased. Median survival in these patients is about 1 month. Treatment options in patients with CNS involvement include intrathecal chemotherapy, radiotherapy, debulking surgery and systemic corticosteroids.^3^ Herein we report a rare case of prostate cancer with leptomeningeal and bone marrow involvement. Although it is reported that the prognosis of these patients is very poor the patient showed a very good response to treatment. After 11 months of follow up, he is alive with excellent performance status. The treatment approach used for the patient might be helpful for the similar clinical scenarios.

## 2 CASE

**FIGURE 1:**
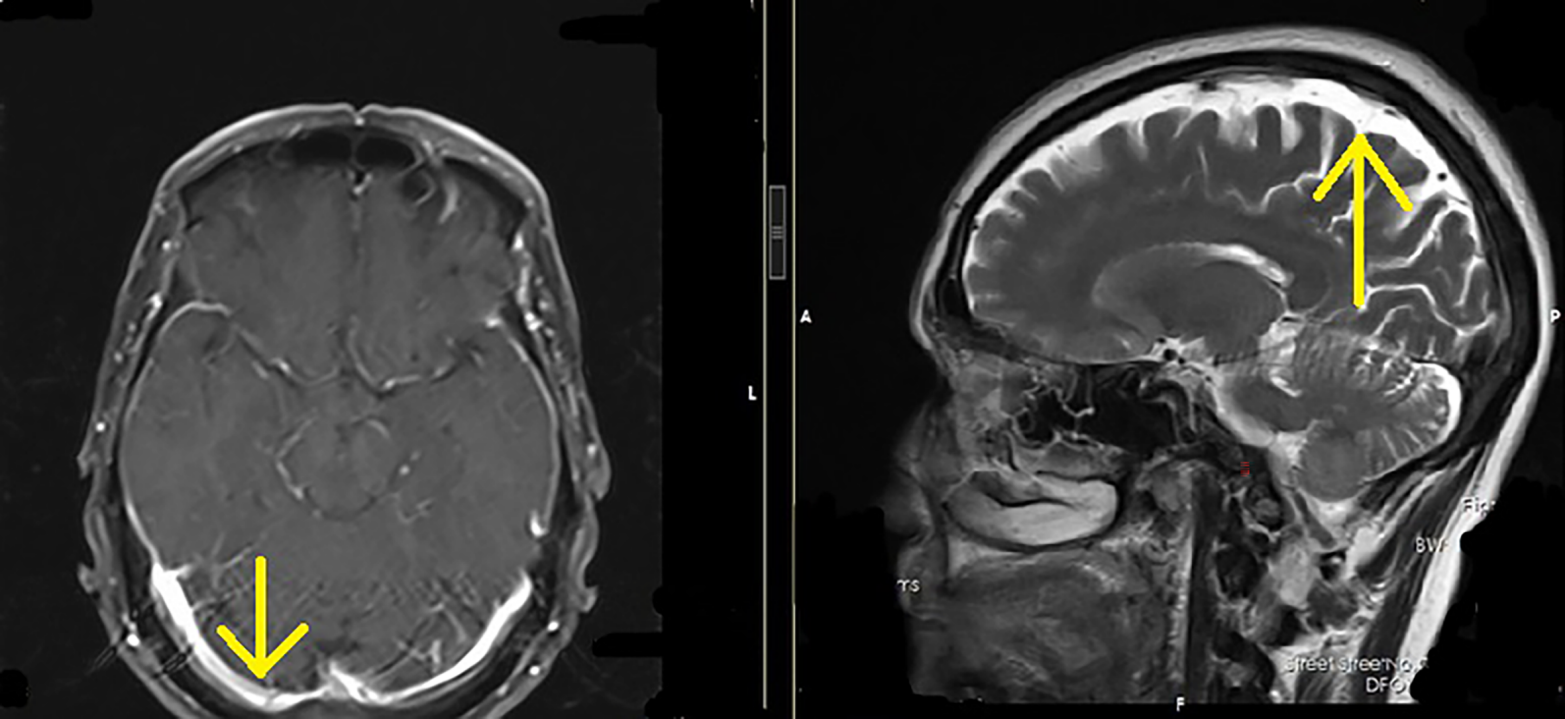
Post‐contrast T1 weighted sequences of the brain MRI at the time of diagnosis of leptomeningeal involvement (April 2022): (A) Axial plane at the level of midbrain and (B) Sagittal planes shows abnormal enhancement in dura (yellow arrows).

**FIGURE 2:**
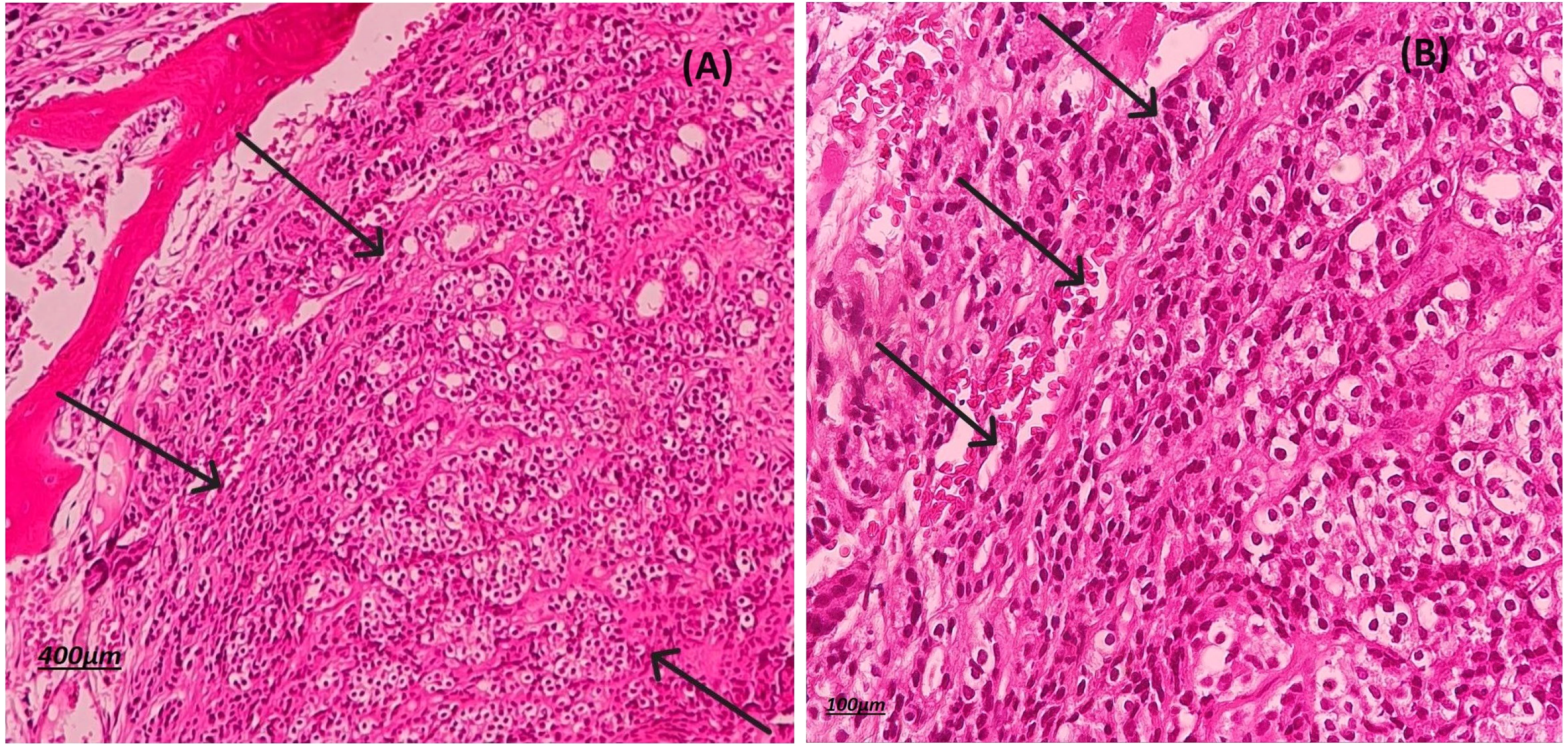
Bone marrow biopsy indicates involvement; hematoxylin and eosin stain demonstrating histologic appearance of the neoplasm: sheets, aggregates, and nests (arrows A: ×100) of atypical epithelial cells (arrows B: ×400) with high Nucleus/Cytoplasmic ratio, clear cytoplasm and prominent nucleoli infiltrated most of bone marrow spaces.

**FIGURE 3:**
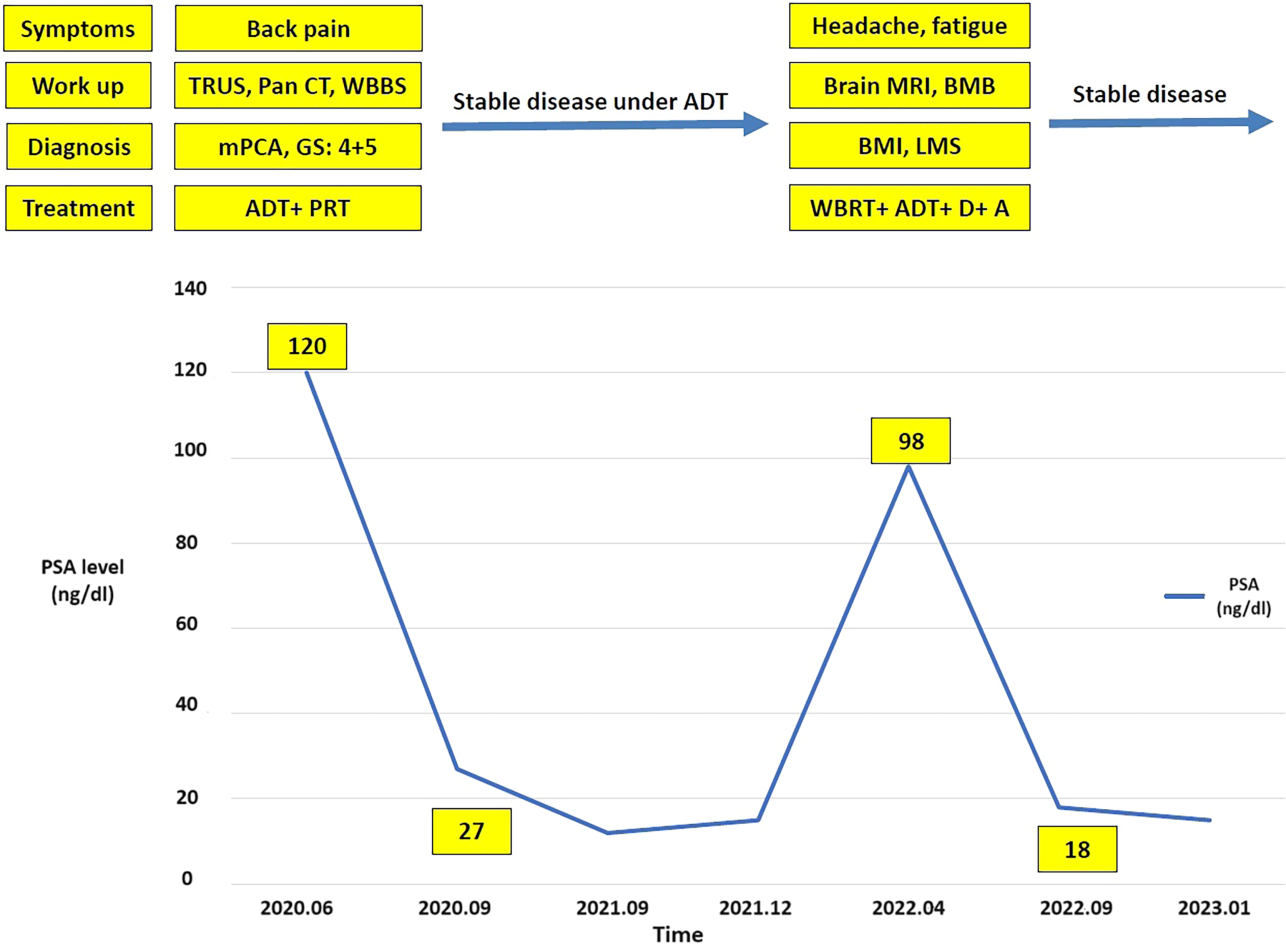
Clinical, biochemical, and treatment course of disease. A, abiraterone; abdomen and pelvis; ADT, androgen deprivation therapy; BMB, bone marrow biopsy; BMI, bone marrow involvement; D, docetaxel; LMS, leptomeningeal seeding; mPCA, metastatic prostate cancer; Pan CT, CT scan of the chest; PRT, palliative radiotherapy; TRUS, trance rectal ultra‐sonography; WBBS, whole body bone scan; WBRT, whole brain radiotherapy.

A 60‐year‐old man presented with back pain and PSA level above 100 ng/mL in laboratory tests referred to radiation oncology ward of Hafte‐tir hospital Tehran Iran in 2020. Core biopsy of prostate showed prostate adenocarcinoma with Gleason score of 4 + 5. A 99mTc‐MDP bone scan and a whole‐body CT scan were ordered to evaluated metastases, which revealed widespread bone metastasis with no evidence of visceral metastases. The patient was treated with leuprolide (11.25 mg each 84 days), bicalutamide (50 mg daily), zoledronic acid (4 mg every 28 days) and palliative irradiation (3000 cGy in 10 fractions) for bone metastases, which resulted in good clinical and biochemical response. Serum PSA level decreased to 27 ng/mL. Chemotherapy and abiraterone acetate was recommended to the patient but refused due to the economic issues. After 2 years of follow up, while receiving leuprolide, progressive headache and fatigue was reported by the patient. The laboratory tests showed rising PSA level to 98 ng/mL despite testosterone level of lower than 20. In complete blood count (CBC), Hemoglobin and platelets level was 8 g/dL and 45 000/μL. Brain MRI manifested diffuse abnormal thickening of meninges with possibility of metastatic meningeal infiltration (Figure 1). Patient was considered to have leptomeningeal carcinomatous according to high level of PSA and MRI findings. Bone marrow aspiration was done due to anemia and thrombocytopenia. Microscopic view showed bony trabeculae with inter‐trabecular marrow tissue replaced by aggregations of atypical epithelial cells, as a result of metastatic carcinoma (Figure 2). Whole brain RT (30 Gy in 10 fractions) was done which resulted in symptoms improvement. The patient performance status according to Eastern cooperative oncology (ECOG) was 1. Patient was treated with combination of docetaxel 50 mg weekly (for 12 cycles), abiraterone 1000 mg daily and prednisolone (10 mg per day). Nplate® and Granulocyte colony stimulating factor (GCSF) were administrated according to laboratory tests. After 5 month of systemic therapy serum PSA level was 18 ng/m and CBC improved (platelet level of 110 000/μL). In the last follow up after 11 months the patients is still symptom free with no rise in PSA level. Clinical, biochemical, and treatment course of disease is shown in Figure 3.

## 3 DISCUSSION

Leptomeningeal metastasis is uncommon in solid tumor and is associated with poor prognosis. The most common tumors that cause leptomeningeal seeding include lung, breast, and melanoma. Leptomeningeal involvement could occur as a result of hematogenous spread, direct extension from bone metastases or along with the perineural lymphatic tract.^2^ However, the CNS is typically not the first site for metastasis in prostate cancer patients. In a study of 31 patients with metastatic prostate cancer to the CNS, all patients had prior metastatic foci to other organs.^4^ Despite the rarity of metastasis to the CNS (brain or the Leptomeninges) in prostate cancer, a study reported in 2011 by Caffo et al. suggested an increased incidence of CNS metastases in the docetaxel era. According to this study, use of docetaxel chemotherapy in patients with castration resistant prostate cancer (CRPC) has led to improved survival and consequently an increased incidence of CNS metastases in these patients. In this study, 943 CRPC patients who were treated between 2002 and 2010 were examined. Of these patients, 31 were reported to have central nervous system metastases (22 with brain metastases and 9 with leptomeningeal metastases). The study revealed that the average survival after the discovery of central nervous system metastases was approximately 4 months.^4^ This is in contrast to older reports, such as a study conducted at MD Anderson in 1999, where the incidence of central nervous system metastases was reported as low as 0.7% in a cohort of 7994 patients over an 18‐year period.^5^ It has even been suggested that the discrepancy between the systemic effectiveness of docetaxel and its low CNS penetration may also contribute to the potential rise in prostate cancer CNS involvement.

Sign and symptoms of patients with CNS involvement might be different depending to the site of involvement. In a retrospective study conducted at MD Anderson in 2012, of 31 patients with genitourinary tumor and leptomeningeal metastases, 19 patients exhibited cerebral symptoms (including seizures, headache, vomiting, confusion), 12 patients presented with cranial nerve symptoms (facial numbness, facial weakness, double vision) and 13 patients presented with spinal cord symptoms (urinary retention, leg weakness).^6^

Diagnosis of leptomeningeal involvement is through CSF cytology test or MRI with contrast.^2^ The best imaging modality for diagnosis of leptomeningeal seeding is gadolinium‐enhanced T1‐weighted MR imaging which has sensitivity and specificity of 70% and 77%–100%, respectively.^7^ Repeating CSF cytology can improve the relatively low sensitivity of this test, from 50% after a single Lumbar puncture to 90% after three times.^3^ However, in the presence of typical clinical finding, MRI is sufficient for the diagnosis.^7^

In a case report in 2008, a 72 years old patient with castration resistant metastatic prostate cancer who was treated with goserelin and docetaxel became lethargic and confused after discontinuation of chemotherapy. In CSF examination of the patient, malignant cells were seen. The patient was treated with two courses of intra‐thecal methotrexate. Despite temporary improvement in clinical symptoms and PSA level, patient was expired due to disseminated intravascular coagulation (DIC) and dyspnea 2 weeks after chemotherapy.^8^ In 2010, a 70 years old prostate cancer patient with involvement of bone, lymph nodes and leptomeningeal seeding in MRI was reported. In CSF cytology no malignant cell was seen although PSA level in CSF was reported to be 6 ng/mL. The patient was treated with docetaxel every 3 weeks and whole brain radiotherapy. Patient's symptoms improved immediately after completion of the treatment, but 3 months later patient expired due to respiratory failure.^9^ Another report in 2012 was related to an 87 years old man with prostate cancer who manifested with leptomeningeal involvement. The Patient received leuprolide as ADT, which led to an improvement in clinical symptoms and decrease in serum PSA level.^10^ In another report in 2019, a 71 years old man with castration resistance prostate cancer and involvement of bone, bone marrow and bladder were treated with denosumab, loperamide, abiraterone and prednisolone. The patient underwent MRI due to lower limb weakness. Leptomeningeal involvement was revealed but due to patient's poor performance, treatment was discontinued.^11^ In 2021, a 69‐year‐old patient with castration resistant metastatic prostate cancer with headache and ataxia was reported. MRI revealed a mass in right occiput, increased meningeal thickness, and diffuse contrast enhancement. Patient treated with ADT and docetaxel. Due to lack of response and rising level of PSA, docetaxel was discontinued, and abiraterone acetate was initiated with whole brain irradiation. The patient expired 1 month later.^12^ As it is evident from the mentioned case reports, there is no specific treatment available for patients with leptomeningeal involvement, and the choice of treatment will depend on the patient's performance status and the opinion of the expert physician. Sometimes, even after being informed about the prognosis, patients choose not to pursue treatment. In a study conducted at MD Anderson between 1978 and 2011, 31 patients with genitourinary tumors and leptomeningeal metastasis were reported. Of whom, 7 patients received brain radiotherapy alone, 5 patients received intrathecal chemotherapy, and 11 patients received both treatments. Additionally, 8 patients did not receive any treatment. There was no significant difference in overall survival among the treatment groups.^6^ As previously mentioned, the prognosis for these patients is very poor. In a systematic review published in 2020, the average interval from meningeal metastases to death was 9 month.^13^

Bone marrow infiltration reported in solid tumor is associated with poor prognosis too. Unlike Bone metastases, bone marrow involvement is not frequent in prostate cancer and is seen in advanced stage of disease. Albeit poor prognosis, there are some reports of good response to systemic treatments such as docetaxel, mitoxantrone and androgen deprivation therapy (ADT).^14^

The number of reported prostate cancer patients with leptomeningeal involvement is limited. However, with more efficient treatment evolving in daily practice and the improvement in the prognosis of metastatic prostate cancer, more patients might be diagnosed with unusual metastases like leptomeningeal disease and the oncologists should consider such a phenomenon in the presence of sign and symptoms. In 2022, a similar case report was published by Dehghani et al., describing a patient with castration resistant prostate cancer who developed bone metastases ~2 years after completing primary treatment. Shortly thereafter, the patient experienced headache and true vertigo, prompting a brain MRI that led to diagnosis of leptomeningeal metastases. In contrast to our report, only palliative whole brain radiotherapy was opted due to the poor prognosis. Although, the patient had previously received abiraterone and docetaxel separately during the course of the disease. The patient passed away 1 month after completing radiotherapy. In our case, various treatment options, potential side effects, and the disease prognosis were discussed with the patient. Finally, a decision was made to pursue triplet therapy (abiraterone, docetaxel and anti‐ androgen) in addition to WBRT, which resulted in a favorable response and an increase in survival.^12^

The main point is that, although the prognosis of the patients with leptomeningeal metastases is poor but there might be some patients who experience a longer survival with new treatments. A noteworthy aspect in this case report is the favorable survival and maintenance of patient performance with triplet therapy despite both leptomeningeal and bone marrow involvement. Triplet therapy (docetaxel and abiraterone added to ADT) have been shown to improve overall survival in de novo metastatic prostate cancer.^15^ Such a strategy might be helpful in patients with poor prognostic factors like the presented case.

## AUTHOR CONTRIBUTIONS

**Maryam Garousi:** Conceptualization (lead); data curation (lead); supervision (equal); writing – original draft (equal). **Nima Mousavi Darzikolaee:** Writing – original draft (supporting); writing – review and editing (lead). **Ali Faridfar:** Data curation (supporting); writing – review and editing (supporting). **Seyed Mohammadreza Javadi:** Data curation (supporting); writing – review and editing (supporting). **Masoumeh Sajadi Rad:** Data curation (supporting); writing – review and editing (supporting). **Esmaeil Samizadeh:** Data curation (supporting); resources (equal). **Reyhaneh Bayani:** Conceptualization (lead); data curation (lead); supervision (equal); writing – original draft (equal).

## CONFLICT OF INTEREST STATEMENT

The authors have stated explicitly that there are no conflicts of interest in connection with this article.

## ETHICS STATEMENT

A written informed consent was taken from the patient for the publication of case details and use of images.

## Data Availability

The data that support the findings of this report are available from the corresponding author upon reasonable request.
